# Plotgardener App: a graphical interface for publication-ready genomic visualization

**DOI:** 10.1093/bioinformatics/btag649

**Published:** 2026-08-28

**Authors:** Rishabh Sharma Vemuri, Abiye Berhanu, Grace E Kenney, Sarah M Parker, Jeffrey T Phillips, J P Flores, Tooba Rashid, Douglas H Phanstiel

**Affiliations:** Department of Computer Science, University of North Carolina at Chapel Hill, Chapel Hill, NC 27599, United States; Department of Computer Science, University of North Carolina at Chapel Hill, Chapel Hill, NC 27599, United States; Curriculum in Bioinformatics and Computational Biology, University of North Carolina at Chapel Hill, Chapel Hill, NC 27599, United States; Curriculum in Bioinformatics and Computational Biology, University of North Carolina at Chapel Hill, Chapel Hill, NC 27599, United States; Department of Computer Science, University of North Carolina at Chapel Hill, Chapel Hill, NC 27599, United States; Curriculum in Bioinformatics and Computational Biology, University of North Carolina at Chapel Hill, Chapel Hill, NC 27599, United States; Curriculum in Bioinformatics and Computational Biology, University of North Carolina at Chapel Hill, Chapel Hill, NC 27599, United States; Curriculum in Bioinformatics and Computational Biology, University of North Carolina at Chapel Hill, Chapel Hill, NC 27599, United States; Thurston Arthritis Research Center, University of North Carolina at Chapel Hill, Chapel Hill, NC 27599, United States; Department of Cell Biology and Physiology, University of North Carolina at Chapel Hill, Chapel Hill, NC 27599, United States

## Abstract

**Summary:**

Plotgardener is an R package used for generating high-quality genomic visualizations. Despite its broad range of functions and versatility, its reliance on code presents a barrier for many potential users. To address this, we developed a macOS desktop application version of Plotgardener that enables users to create publication-ready genomic plots with no programming experience. The application employs a modular architecture comprising an Electron.js backend, a React frontend, and a Python parser that dynamically analyzes the Plotgardener package to ensure interface fields remain synchronized with package updates. By lowering the technical barrier to advanced genomic visualization, the Plotgardener desktop application broadens access to powerful visualization workflows for researchers and clinicians.

**Availability:**

The current release of the Plotgardener App is an open source macOS desktop application built with Electron (Node.js), featuring a React frontend and a Python-based parser. The download link is available at https://phanstiellab.github.io/plotgardener/articles/guides/plotgardenerApp.html and on Zenodo (doi: https://doi.org/10.5281/zenodo.21684228). The source code is hosted on GitHub at https://github.com/rishabhsvemuri/ThePlotgardenerApp.

## 1 Introduction

Plotgardener (Kramer *et al.* 2022) is an R package for the generation of flexible, multipanel, publication-quality visualization of genomic and non-genomic data that has been downloaded over a thousand times and cited in over 100 papers. Despite these advantages, using Plotgardener requires proficiency with the R programming language (R Core Team 2021), R package management, and basic terminal commands. Many biologists may not have this experience, posing a high barrier to entry for using Plotgardener to present their research. While multiple point-and-click browsers (Kent *et al.* 2002, Carver *et al.* 2009, Flicek *et al.* 2011, Robinson *et al.* 2011, Abeel *et al.* 2012, Chelaru *et al.* 2014, Durand *et al.* 2016, Kerpedjiev *et al.* 2018, Seng *et al.* 2025) are available for easy and fast data browsing, these tools are optimized for browsing, whereas Plotgardener is designed for flexible, publication-ready figure assembly. Here, we introduce the Plotgardener App, a macOS graphical desktop interface for Plotgardener that allows users to generate publication-quality genomic visualizations without requiring programming experience. The interface supports assembly of multipanel genomic figures, data import, annotation, session saving, and export of editable R code.

## 2 The Plotgardener App

We present the Plotgardener App, a macOS desktop application that brings Plotgardener functionality to a graphical user interface.

### 2.1 Installation

To minimize installation and setup effort on macOS, the Plotgardener App is distributed as a single .dmg disk image. The disk image contains the packaged Electron application, including the React-based user interface and bundled application resources. Users install the app by opening the .dmg file and moving the application into the Applications folder.

### 2.2 Usage

**Figure 1:**
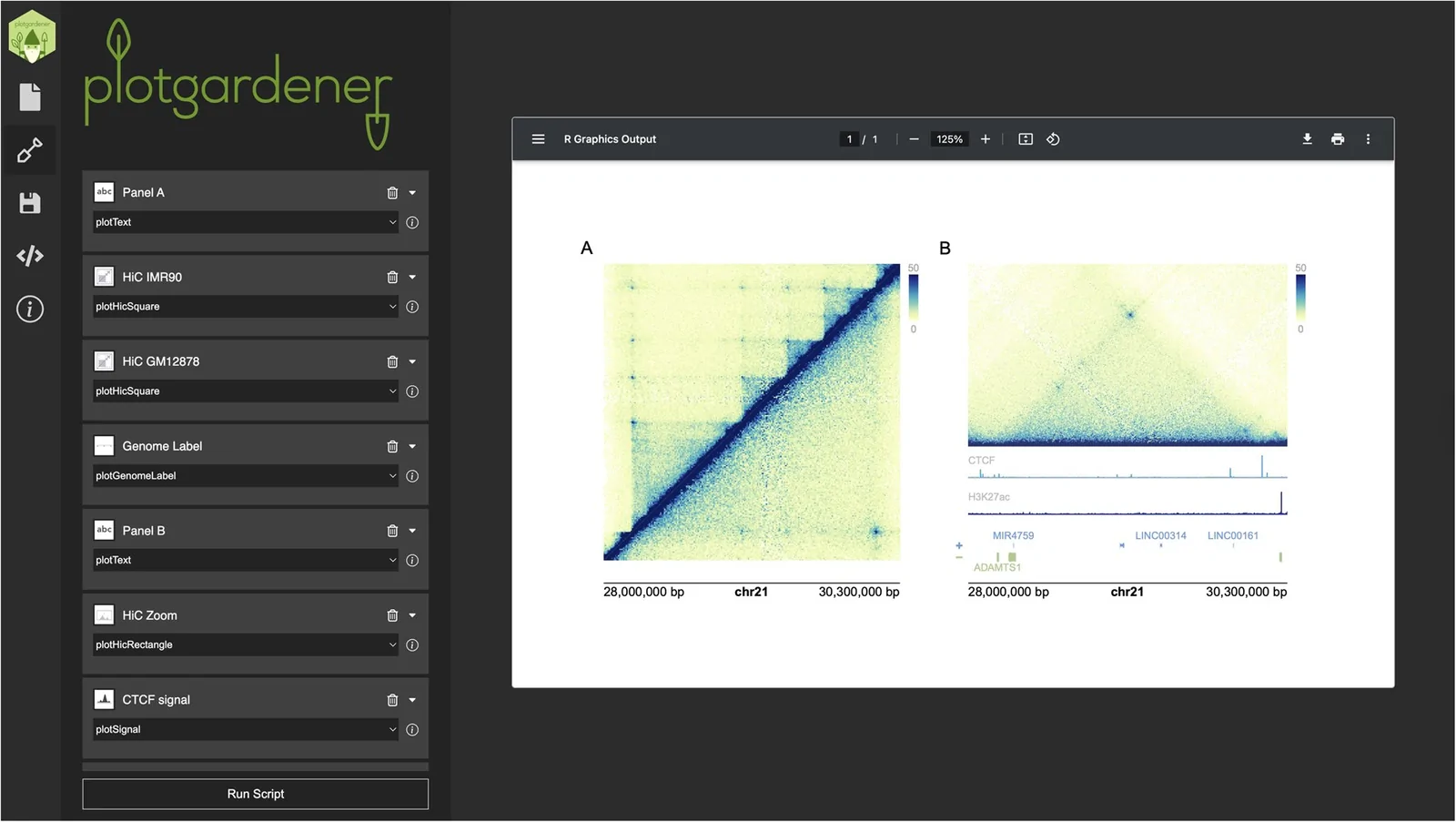
Screenshot of the Plotgardener App displaying a multipanel genomic visualization with Hi-C and ChIP-seq data tracks.

The application is organized through a series of tabs displayed on the left side of the application window (Fig. 1), which guide users through the process of building a visualization. The “Create Page” tab allows users to define global figure attributes such as page size and units. The “Plot Addition” tab enables users to add and configure multiple plots of supported types through a dropdown-based interface, with options for data upload, parameter specification, and annotation. Each plot can be customized independently, including the addition of annotations layered on top of the visualization. The “Save” tab allows users to export and import sessions as structured JSON files, enabling the reuse of figure templates and sharing across different machines. The “Code” tab displays the R code generated by the application, allowing advanced users to copy and further modify it in an external development environment. Once configured, users can execute the visualization workflow, and the resulting figure is rendered directly within the application.

### 2.3 Implementation

The application is built with Electron and consists of three components: frontend, backend, and parser.

The frontend is implemented as a set of React components within Electron that dynamically generate the interface from structured JSON definitions parsed from the Plotgardener package. Rather than hard-coding forms, the Create Page and Plot Addition tabs render inputs at runtime based on function parameters, including defaults, options, and validation rules. User inputs are synchronized with backend data structures through Electron bridge methods, allowing plots and annotations to be added, modified, and reordered while preserving the structure needed for R script generation. The Save component serializes the full application state, including plots, annotations, and backend data, into session files that can be reloaded to reconstruct the interface. The Code component displays and updates the generated R script, while the Output component listens for execution events and updates the rendered PDF, loading state, or error messages accordingly. Together, these components form a reactive pipeline that transforms package-derived definitions into user inputs, R code, and final visualizations.

The backend layer stores and manages user-specified plotting parameters from the frontend. Inputs can be added, edited, reordered, or deleted while preserving the structure required for R script generation. When the user clicks “Run Script,” the backend compiles the stored inputs into an executable R script and runs it in a separate bash session. The resulting PDF is then stored and displayed in the application, and the generated R script is returned to the frontend for user review and modification.

The parser layer maintains a dynamic connection to the source Plotgardener R package. Supported functions, parameters, defaults, and comments are parsed into structured JSON files that are used by the frontend to generate the corresponding input fields. This design allows updates to the underlying package to be reflected in the interface by regenerating the JSON files used by the frontend layer.

## 3 Conclusions

We developed the Plotgardener App, a macOS graphical desktop interface for Plotgardener that enables users to generate publication-ready genomic visualizations without requiring programming experience. By coupling the interface to a parser that dynamically extracts functions and parameters from the underlying R package, the application lowers the barrier to figure generation while remaining synchronized with ongoing package development. This framework broadens access to Plotgardener and provides a general strategy for extending code-based scientific software through maintainable graphical interfaces.

## Data Availability

Plotgardener App installation instructions, documentation, and sample data used to generate the figure in this paper can be downloaded from the Plotgardener website (https://phanstiellab.github.io/plotgardener/articles/guides/plotgardenerApp_getting_started.html). Source code for the Plotgardener App is available on GitHub (https://github.com/rishabhsvemuri/ThePlotgardenerApp).
